# *In situ* changes of tropical crustose coralline algae along carbon dioxide gradients

**DOI:** 10.1038/srep09537

**Published:** 2015-04-02

**Authors:** K. E. Fabricius, A. Kluibenschedl, L. Harrington, S. Noonan, G. De'ath

**Affiliations:** 1 Australian Institute of Marine Science, PMB 3, Townsville Qld 4810, Australia; 2 Alfred Wegener Institute, Helmholtz Centre for Polar and Marine Research, D-27568 Bremerhaven, Germany; 3 James Cook University, School of Marine and Tropical Biology, Townsville, Qld 4811, Australia

## Abstract

Crustose coralline algae (CCA) fulfill important ecosystem functions in coral reefs, including reef framework stabilization and induction of larval settlement. To investigate *in situ* the effects of high carbon dioxide on CCA communities, we deployed settlement tiles at three tropical volcanic CO_2_ seeps in Papua New Guinea along gradients spanning from 8.1 to 7.4 pH. After 5 and 13 months deployment, there was a steep transition from CCA presence to absence around pH 7.8 (660 μatm *p*CO_2_): 98% of tiles had CCA at pH > 7.8, whereas only 20% of tiles had CCA at pH ≤ 7.8. As pH declined from 8.0 to 7.8, the least and most sensitive CCA species lost 43% and 85% of cover, respectively. Communities on upward facing surfaces exposed to high light and high grazing pressure showed less steep losses than those on shaded surfaces with low grazing. Direct CO_2_ effects on early life stages were the main mechanisms determining CCA cover, rather than competitive interactions with other benthic groups. Importantly, declines were steepest at near-ambient pH, suggesting that CCA may have already declined in abundance due to the recent seawater pH decline of 0.1 units, and that future severe losses are likely with increasing ocean acidification.

Crustose coralline algae (CCA, family Corallinaceae, Rhodophyta^1^) play an important role in many marine ecosystems, providing reef framework, shore protection and carbonate sediments in shallow water^2^, and facilitating the settlement and survival of larvae of numerous other benthic taxa^3^,^4^,^5^,^6^. The cell walls of CCA are heavily calcified with high-magnesium calcite^7^,^8^, which is more soluble than other forms of calcium carbonate at low pH^7^. CCA are therefore considered highly vulnerable to ocean acidification (OA), i.e., the rapid global decline in surface seawater pH and associated changes in the seawater carbonate chemistry from rising atmospheric CO_2_. Several laboratory and mesocosm studies have documented negative effects of OA on CCA calcification, dark dissolution and bleaching^9^,^10^, recruitment^11^, survival^12^, and rates of morphological abnormalities and mortality in newly settled CCA^13^. Field studies have also documented severely reduced CCA cover at elevated CO_2_ at both temperate and tropical volcanic CO_2_ vents, and other settings with contrasting seawater pH^14^,^15^,^16^,^17^,^18^.

Information on differences in the OA susceptibility between individual CCA species is limited, despite their diverse ecological functions, including great inter-specific differences in their effectiveness as reef framework builders^19^, and as settlement substrata for coral larvae^3^,^4^,^6^. One study showed that in tropical Indo-Pacific CCA, thin encrusting and early successional species, which are particularly important as settlement substrata, appear to be more OA-sensitive than more robust taxa with thicker crusts^6^. Another study showed that in temperate Northeastern Pacific CCA, two species that form thick crusts have become substantially thinner over the last 20 years, whereas two thinner species maintained similar crust thickness but reduced the thickness of some of their cell walls^20^. Others have documented an unusually high CO_2_ tolerance in thick crusts of the tropical *Porolithon onkodes*, and suggested that this common species will likely continue to provide essential reef and shore protection in future oceans under OA^8^,^21^. An improved understanding of the different responses of tropical CCA taxa to OA is therefore warranted, to improve predictions about the future of these communities and the ecosystem services they provide.

To facilitate such predictions, estimates of the *in situ* response curves of CCA species to increasing CO_2_ are needed. Biological responses to environmental stressors are often slow and non-linear, hence the choice of stressor concentrations and exposure times in laboratory experiments can strongly influence conclusions about the direction and magnitude of change^22^,^23^. Two laboratory studies that investigated the relationships of CCA along pH gradients found linear relationships and no apparent tipping points in CCA growth and survival^9^,^13^. However, laboratory experiments are unlikely to reflect ecosystem responses in the field, where a multitude of biotic and environmental factors including light, currents, waves, sedimentation, temperature, recruitment limitation, competition, grazing and acclimatization co-determine CCA abundances^19^.

There is also conflicting information about the ecological mechanisms that determine how OA will affect CCA. Two experimental studies identified recruitment limitation as the main mechanism of change at near-future levels of OA^11^,^13^. In contrast, an *in situ* study at a temperate rocky shore Mediterranean CO_2_ vent system concluded that low CCA cover was the outcome from shifts in competitive advantage at reduced pH, while rates of recruitment remained unaltered^17^.

The objective of this study was to compare the response curves and tolerance thresholds of various CCA taxa to OA, and identify the likely underlying mechanisms for their responses, *in situ* in coral reefs under both high and low light and grazing pressure. The study was conducted at three volcanic CO_2_ seeps in Papua New Guinea, which have exposed the reef to additional CO_2_ for a confirmed 70 years, and possibly much longer^15^. First, we assessed the cover of CCA in the natural reef communities at the CO_2_ seeps and at nearby control sites. Second, we investigated the cover of various CCA taxa and of their space competitors on settlement tiles deployed along CO_2_ gradients for 5 and 13 months. The study was conducted at three CO_2_ seep sites and three adjacent control sites away from the seeps, at 3 m depth in clear tropical waters. The CCA communities at the seep and control sites were exposed to similar natural conditions of flow, light, waves, sedimentation and temperature, and natural levels of grazing and competitive interactions with other biota. Differences in the communities on the upward and downward facing surfaces of the tiles were used to compare pH responses in high and low light, and under high and low grazing pressure from macro-grazers. The analysis of these data illustrate potential ongoing and future effects of increasing OA on the ecology of common CCA taxa in the tropical Indo-Pacific, and identify potential tipping points in their pH tolerances.

## Results

### Seawater chemistry

For the reef transects at the three Control sites, the median pH (total scale) ranged from pH 8.02 to 7.98 units and *p*CO_2_ ranged from 346 to 413 μatm. At the High CO_2_ sites, median seawater pH values ranged from 7.95 to 7.72, and calculated *p*CO_2_ concentrations ranged from 441 to 998 μatm^24^.

**Figure 1:**
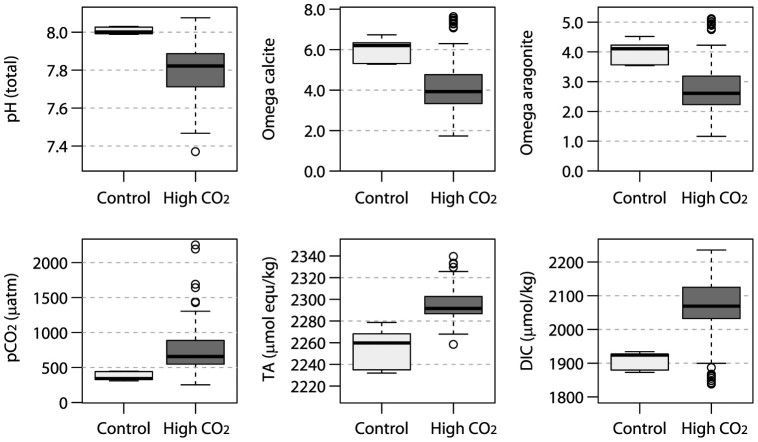
Seawater chemistry over the Control and High CO_2_ tiles. Black horizontal lines indicate medians; boxes enclose the upper and lower quartiles of the data, whiskers mark the maximum and minimum values excluding outliers, while round circles show outliers. The plots show the median values of 45 Control and 71 High CO_2_ tiles, with each of these 45 and 71 median values composed of 4 to 23 measurements taken directly over each of the tiles during four two-week long visits in the period 2011–2012 (data are listed in the Supplementary Tables S1 and S2, online). pH is at total scale, Omega calcite and aragonite = saturation states of calcite and aragonite, TA = total alkalinity, DIC = dissolved inorganic carbon.

The individual tiles were exposed to seawater chemistry conditions ranging from median values of pH 8.08 to 7.37, 1838 to 2236 μmol kg^−1^ dissolved inorganic carbon (DIC), and saturation state of calcite (Ω_Calc_) of 7.6 to 1.73 (Fig. 1, Supplementary Table S1). Differences in the six seawater chemistry parameters between seep and control sites were large and highly significant (Supplementary Table S2). For the tiles from the Control sites, the median pH averaged 8.01 (5^th^ and 95^th^ percentiles: 7.95, 8.09) and the median *p*CO_2_ was 397 μatm (300, 484). For the High CO_2_ tiles, pH averaged 7.80 units (7.52, 7.97), with a median *p*CO_2_ concentration of 660 (492, 1128) μatm, total alkalinity (TA) was elevated by ~30 μmol equivalents kg^−1^ (median: 2292 μmol equivalents kg^−1^) and dissolved inorganic carbon (DIC) by 140 μmol kg^−1^ (median: 2069 μmol kg^−1^). Tiles at the Control sites were exposed to a median Ω _Calc_ of 6.20 (5.29, 7.41), while at the High CO_2_ sites Ω_Calc_ was 3.93 (2.28, 6.73). Similarly, the median saturation state of aragonite (Ω_Arag_) was reduced from 4.11 (3.55, 4.97) at the Control sites to 2.61 (1.53, 4.52) at the High CO_2_ sites. Of all tiles, 42% were exposed to a median Ω_Arag_ ≤ 3.0, a value commonly assumed a threshold for reef development^25^. The median Ω_Arag_ for all tiles was >1.0, but 10% of the tiles were exposed to Ω _Arag_ ≤ 1.0 for up to 5% of their time.

### Comparison of CCA between the High CO_2_ and Control sites

**Figure 2:**
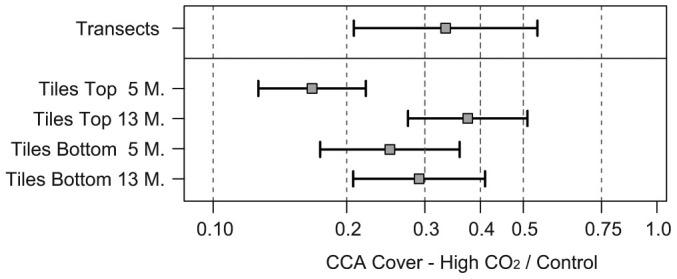
Log ratios of the cover of crustose coralline algae (CCA) at High CO_2_ over Control sites, on the three reefs (Reef Communities, N = 80 transects), and on the top- and bottom-sides of the settlement tiles after 5 and 13 months of deployment (5.M, 13.M; N = 120 and 116, respectively). Squares indicate back-transformed means, the error bars are 95% confidence intervals (Supplementary Table S3). For example, the ratio 0.3 indicates the mean cover at High CO_2_ is 30% of that at the Controls. Differences are all significant at the 5% level (error bars do not include the value 1.0).

Along the transects on the reef, total CCA cover at the Control sites was ~3-fold higher than at the High CO_2_ sites, with 6.65% (95% confidence intervals: 4.43%, 8.53%) vs. 2.22% (1.48%, 3.34%); (Fig. 2, SI Table S3). Unoccupied space, i.e. space covered only by biofilms or very sparse turf algae, was ~30% and 24% of natural reef surfaces at the Control and High CO_2_ sites, respectively.

**Figure 3:**
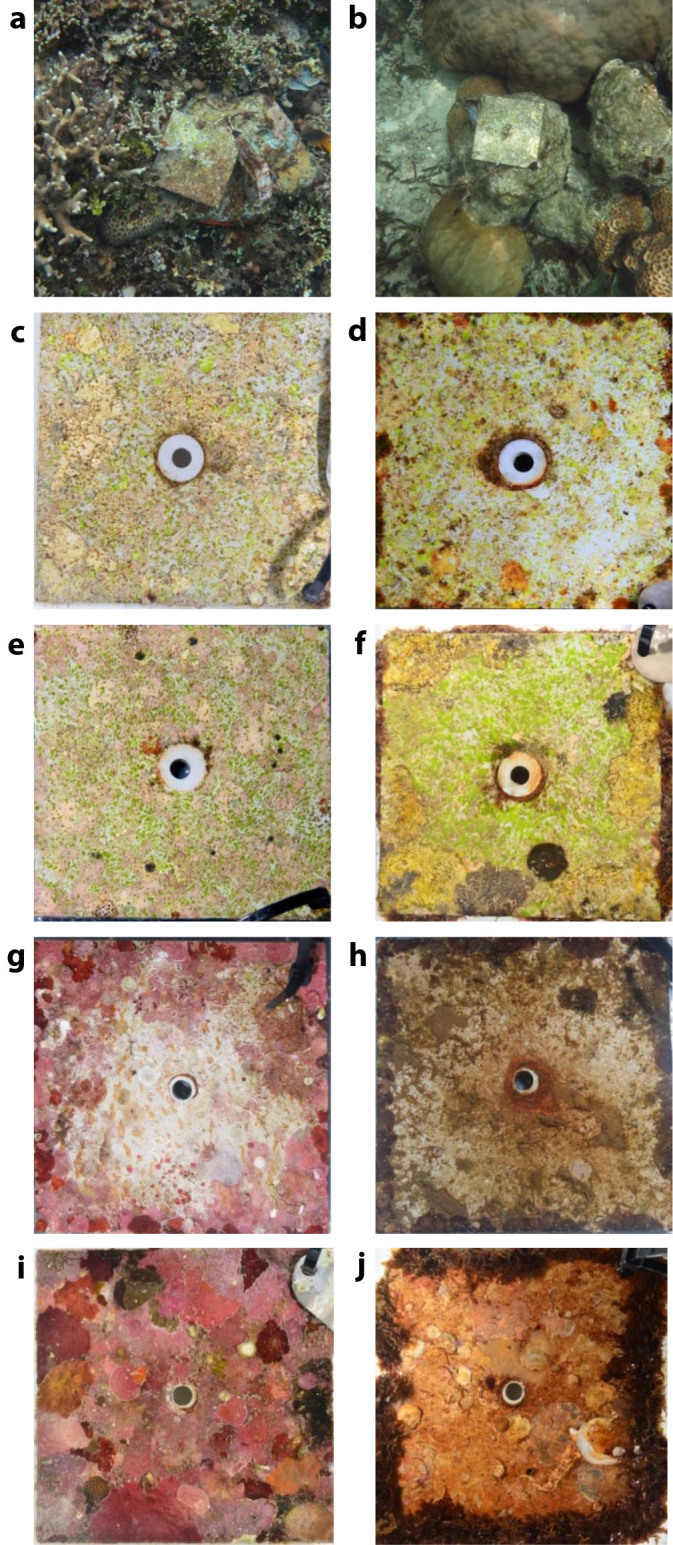
CCA settlement tiles at Control sites (left) and High CO_2_ sites (right) at volcanic CO_2_ seeps in Papua New Guinea. Tiles *in situ* after 13 months deployment (a, b). Top sides of tiles after 5 months deployment (c, d), and after 13 months (e, f). Bottom sides of the tiles after 5 months (g, h), and after 13 months (i, j).

On the top-sides of the tiles, total CCA cover also responded strongly to CO_2_. After 5 months of deployment, total CCA cover was ~6-fold higher on the Control compared to the High CO_2_ tiles, with cover of 38% (29.7%, 43.3%) vs 6.4% (5.0%, 8.15%); (Figs. 2, 3, Supplementary Table S3). After 13 months, this difference was reduced to 2.7-fold, with 29.6% (23.1%, 35.6%) vs 11% (8.65%, 14.2%), due to a reduction in CCA cover at the Control and slight expansion at the High CO_2_ sites. Total CCA cover at the 13 month census was significantly correlated to that at the 5 months census (correlation coefficient R^2^ = 0.50, F_(1,63)_ = 64.6, P < 0.0001). The amount of unoccupied space on the Control tiles declined slightly (from 23% to 17%) between the two censuses, despite slight reductions in CCA and green filamentous algae, due to small expansions of turf algae, macroalgae and cyanobacteria. Unoccupied space on the High CO_2_ tiles declined substantially, from 36% to 12%, due to the expansion of turf algae, cyanobacteria, CCA and *Peysonellia*. Most top-sides of the tiles, at both the Control and High CO_2_ sites, showed dense patterns of recent scrape marks, and macroalgae were restricted to the tile edges, suggesting exposure to intense grazing by macrograzers including fishes and sea urchins (Fig. 3).

On the bottom-sides of the tiles, after 5 months of deployment, total CCA cover was ~4-fold higher on the Control compared to the High CO_2_ tiles, with 23.5% (17.3%, 28.5%) vs 5.9% (4.33%, 7.95%). After 13 months, the difference between Control and High CO_2_ tiles was 3.4-fold, with 21.4% (16.1%, 25.9%) vs 6.2% (4.69%, 8.26%). As with the top-sides of the tiles, CCA cover on the bottom-sides was significantly correlated between the two census dates (R^2^ = 0.39, F _(1,111)_ = 69.3, P < 0.0001). Unoccupied space declined between the two censuses, from 21% to 7.6% on the Control tiles, and from 20% to 4.4% at the High CO_2_ tiles. The reduction in unoccupied space at both Control and High CO_2_ tiles was due to the expansion of many types of benthic algae and other groups throughout the observation period, while CCA cover remained similar.

### Changes in CCA along CO_2_ gradients

**Figure 4:**
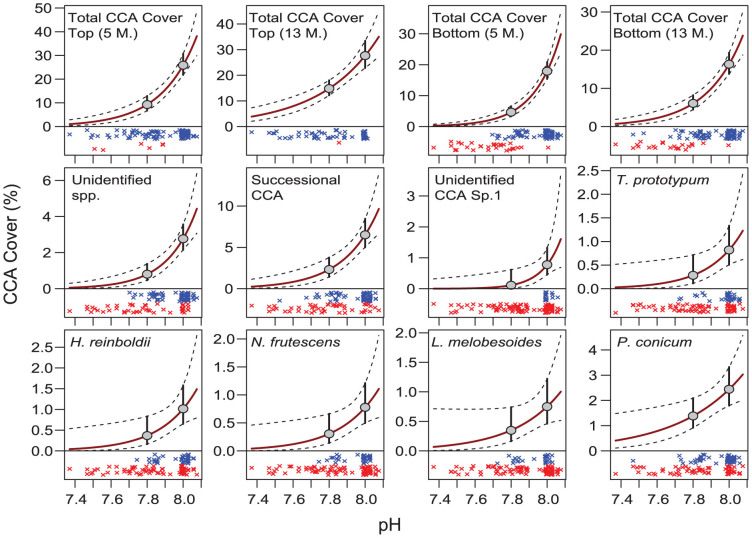
Changes in the cover of various taxonomic groups of CCA along the pH gradient (Supplementary Table S4). Top row: total CCA cover (all CCA taxa combined), on the top- and bottom-sides of the tiles after 5 and 13 months deployment (5 M., 13 M.; N = 120 and 116, respectively). Middle and bottom row: changes in cover of specific CCA taxa on the bottom-sides of the tiles after 13 months. The red solid lines show the estimated cover as a function of pH, dashed lines show upper and lower 95% confidence intervals. The grey dots and vertical bars show mean cover at a pH level of 8.0 and 7.8, and the 95% CI of these estimates. The ‘x’ symbols (jittered vertically for clarity) show the pH of the individual tiles on which the specific CCA taxa were present (blue) or absent (red).

Sorting the tile communities by their median pH levels showed that total CCA cover and the cover of most specific CCA taxonomic groups declined steeply and non-linearly as pH declined from pH 8.1 to 7.4 (Fig. 4). On the top-sides of the tiles, total estimated CCA cover at a median pH of 7.8 (median *p*CO_2_ = 660 μatm, Ω_Arag_ = 2.76) was 36% and 54% of that at pH 8.0 (*p*CO_2_ = 404 μatm, Ω_Arag_ = 4.05) after 5 and 13 months, respectively (Supplementary Table S4). Communities were dominated by *Porolithon* ( = *Hydrolithon*) *onkodes* with a >98% contribution to total CCA cover, and hence the estimates of change of total CCA cover on the top-sides largely reflect the response curves of this single species. Its estimated median cover on the 13 months old tiles declined from 27.6% (95% confidence intervals: 22.9%, 33.4%) at pH 8.0 to 14.8% (12.2%, 18.0%) at pH 7.8, and to 4.3% (0.9%, 20%) at pH 7.4. On the bottom-sides of the tiles, CCA declined even more severely, with gradients being much steeper at the high end of the pH range (Fig. 4, Supplementary Table S4). Here, estimated median cover at pH 7.8 was only 26% and 37% of the cover at pH 8.0 after 5 and 13 months, respectively. On the 13 month old tiles, estimated cover was 16.3% (13.8%, 19.3%) at pH 8.0, 6.1% (4.4%, 8.2%) at pH 7.8, and 0.08% (0.02%, 0.40%) at pH 7.4. The species *Titanoderma prototypum*, *Hydrolithon reinboldii*, a branching species resembling *Neogoniolithon (Spongites) frutescens* (taxonomic identification pending), an unidentified species (Unidentified sp.1), and successional CCA were most severely reduced, with 60% to 85% losses of cover at pH 7.8 vs 8.0. The most pH tolerant taxa were *Lithoporella melobesioides* and *Paragoniolithon conicum*, which lost 53% and 43% of cover respectively. Estimates for *P. onkodes* and *Lithophyllum kotschyanum* from the bottom-sides of the tiles were unreliable due to their rarity on these shaded surfaces (*P. onkodes*: 6 occurrences, all at pH ≥ 7.79; *L. kotschyanum*: 5 occurrences, all at pH ≥ 8.0).

The bottom-sides (low light, little grazing) of the tiles not only had lower CCA cover than the top-sides (high light, intense grazing), but also experienced steeper losses than the top-sides along the pH gradient (Fig. 4, Table 1, Supplementary Table S3). Differences in CCA cover between the two censuses were minor, as the changes along the pH gradient were only marginally weaker after 13 months compared to 5 months.

**Figure 5:**
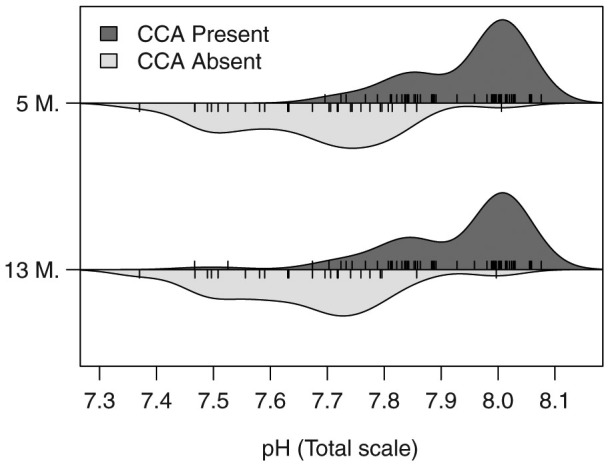
Violin plot showing changes in the frequency of settlement tiles with CCA present on their bottom-sides, along the pH gradient. The dark and light grey areas indicate the densities of observations of tiles with and without CCA, respectively, both after 5 month (5 M.: N = 120) and after 13 month of deployment (13 M.: N = 116). The rugs indicate the median pH for each tile, i.e. the pH to which the tiles were exposed to in the field. The transition from CCA presence to absence is steep at a pH of ~7.8.

The different responses to CO_2_ between top- and bottom-sides of the tiles were even more pronounced when using the measure of CCA presence or absence on the tiles. On the top-sides of the tiles, CCA was present on most tiles, even at the lowest pH, with 92% and 96% of tiles having at least some CCA after 5 and 13 months, respectively (Fig. 4). On the bottom-sides of the tiles, CCA was also present on all tiles from the Control sites, and 96% and 98% of tiles at a median pH > 7.8 had at least some CCA after 5 and 13 months, respectively (Figs. 4, 5). However, there was a steep transition from CCA presence to absence at around pH 7.8: at a pH of ≤7.8, only 20% of the tiles had some CCA, and at pH ≤ 7.7, only 3% of tiles had CCA. The proportion of tiles with and without CCA was very similar between the 5 and 13 months censuses (Fig. 5).

## Discussion

This field study documents strong, non-linear negative relationships between the cover of crustose coralline algae (CCA) and CO_2_ concentrations on both natural coral reef substrata and on settlement tiles. In shaded conditions, CCA cover was reduced to almost zero at a median pH < 7.7, and none of the CCA taxa were resilient to exposure to high CO_2_. The most robust and most sensitive of taxa lost 43% and 85% of cover respectively, as the pH declined from 8.0 to 7.8 units. Importantly, the response curves also suggest steep declines between pH 8.1 and 8.0, and thus many CCA species may have already lost cover as a consequence of anthropogenic CO_2_ emissions, which have lowered the surface seawater pH by 0.1 units compared to pre-industrial times^23^.

Our results from the colonisation of settlement tiles shed light on the potential underlying mechanisms determining the high CO_2_ sensitivity of CCA. The amount of unoccupied space on the top-sides of the 5-month old tiles (23% at Control and 36% at High CO_2_ tiles) was similar to the surrounding reef benthos (24% at Control and 30% at High CO_2_ sites). CCA cover on the top-sides was 6-fold reduced on the High CO_2_ compared with the Controls tiles, although the former had 50% more unoccupied space than the latter. On the bottom-sides, the amount of unoccupied space was similar on High CO_2_ and Control tiles, yet CCA cover was 4-fold reduced. Between the 5 and 13 month censuses, CCA cover gradually increased at High CO_2_ (6.4% to 11%) but declined at the Control tiles (from 38% to 30%). Thus, in early successional stages, competition by other algal groups appeared to be weaker rather than stronger at elevated CO_2_, indicating that the reduced CCA cover at High CO_2_ cannot be attributed to increased competition. As CCA cover at 5 month was well correlated with CCA cover at 13 month, is seems that CO_2_ directly affected CCA life histories within the first 5 months (recruitment, net growth balancing calcification and dissolution, and/or survival), and that it was these factors that largely determined the later CCA cover.

Our finding of direct effects of CO_2_ on early CCA life history factors, rather than CO_2_ effects on later competitive outcomes, contrast with those from settlement tiles deployed at the temperate Mediterranean CO_2_ seeps^17^. In the latter, calcareous species including CCA occupied a similar amount of space (<15%) on top-sides of the tiles within the first 3.5 month at both ambient and elevated CO_2_. After 6.5 and 14 month, CCA cover had not further increased at elevated CO_2_ due to space occupancy and overgrowth by fleshy macroalgae, while at the control sites CCA cover continued to expand to ~25%, suggesting that shifts in competitive advantages rather than early recruitment determined changes in these CCA communities at elevated CO_2_. However, our finding of a bottleneck for early CCA life history stages at high CO_2_ agrees with the results of three other studies. In particular, a 7-week mesocosm study on tropical Indo-Pacific CCA showed a 78% and 92% decline in CCA recruitment and cover respectively, as pH was reduced by 0.26 units from control conditions (an increase in *p*CO_2_ from 400 to 765 μatm)^11^. Similarly, reef-associated CCA species with rapid growth and thin thalli had reduced cover at 400 compared to 800 or 1300 μatm CO_2_, while CCA taxa with thicker crusts were more CO_2_ resistant^6^. In that study, the decline in CCA coincided with higher unoccupied surfaces at elevated CO_2_, suggesting that direct CO_2_ effects on the early life history stages, rather than space competition, were responsible for those losses. Young settlers of the cold water CCA species *Phymatolithon lenormandii* also displayed significantly impaired recruitment success at slightly elevated CO_2_ within four weeks, due to increased mortality and abnormal development^13^. This was due to high rates of both dissolution and regrowth over the whole CCA thallus surface at higher CO_2_, despite similar rates of extension along the growth margins at all pH levels. Finally, Diaz-Pulido *et al.* (2014) showed that in *P. onkodes* the mineralogy of deeper skeletons, but not of the actively growing pink surface layers, can change from high magnesium calcite to the less soluble dolomite under OA^21^. This suggests that such passive change in mineralogy can protect mature established crusts of CCA, but possibly not thin early growth phases. Hence, these studies in combination with ours consolidate the evidence that CO_2_ predominantly affects early CCA life stages, and that these effects on the early life stages co-determine CCA cover in later successional phases. This conclusion is perhaps not surprising, since CCA sporelings are likely to be particularly sensitive to elevated CO_2_, with early calcification being essential for the attachment and integrity of the initially very thin films of (<500 μm) hypothallial filaments^19^. But this finding also suggests that *p*CO_2_ perturbation experiments on established CCA crusts may severely underestimate the CO_2_ effects on future CCA populations, due to the apparent bottleneck of high OA vulnerability in their early life stages.

Grazing intensity may partially explain the different findings about successional mechanisms between the Mediterranean and the PNG seeps study. The former study, which assessed only the top-sides of the tiles in the heavily fished Mediterranean Sea, found that CCA were outcompeted by macroalgae over time^17^. In our study, the top-sides were heavily scraped indicating exposure to intense grazing that likely prevented macroalgal overgrowth. Two grazing sea urchins (*Diadema* spp. and *Echinometra* spp.) are more abundant at the High CO_2_ sites^24^, however communities of grazing fishes vary little between the High CO_2_ and Control sites^26^. Grazing is considered essential to protect some CCA against overgrowth^19^, however some taxa have other means to prevent overgrowth, e.g. by regularly sloughing off surface cell layers, and over-intense grazing can also damage CCA. A laboratory study showed that 21 days of exposure to doubled concentrations of CO_2_ increased the vulnerability of the CCA *Hydrolithon* ( = *Porolithon*) *onkodes* to grazing damage from sea urchins, as CO_2_ reduced the structural integrity of the CCA cell walls^27^. On the bottom-sides of our tiles, it is likely that light limitation prevented the overgrowth by turfs or fleshy macroalgae.

Light limitation may further contribute to determining CCA responses to rising CO_2_. The tiles were deployed at 3 m depth in clear tropical waters, where upward facing surfaces can be exposed to daily irradiances >70% of that at the water surface^28^. The downward facing sides mimic the light environments in cave entrances and under overhangs, where daily irradiance can be reduced by two orders of magnitude compared to the upward facing reef surfaces^28^. The declines in CCA cover in response to CO_2_ were steeper on these shaded bottom-sides compared to the top-sides of the tiles, suggesting that CCA may be physiologically more vulnerable to high CO_2_ in low compared to high light environments. Previous physiological studies have shown that some CCA can continue to grow at high CO_2_ in high light environments, but suffer dissolution in the dark^29^,^30^. Our data also show the need for high light to maintain populations at high CO_2_ in the field, with day-time calcification offsetting potential night-time dissolution. Long-term controlled field and mesocosm experiments are needed to better predict the complex interactions between CO_2_, light and grazing for key tropical CCA species and ecosystems.

Species-specific CO_2_ tolerances may also be contribute to the different responses, as the top sides of the tiles are dominated by *P. onkodes*, while the bottom sides had mixed communities. Previous studies have identified mature crusts of *P. onkodes* as particularly OA tolerant due to the formation of relatively insoluble dolomite in their cell walls^8^,^21^. However, we were not able to confirm an unusually high OA tolerance of young *P. onkodes* on our tiles. On the top-sides *P. onkodes* lost an estimated 52% of cover as pH declined from 8.0 to 7.8, and on the bottom-sides *P. onkodes* was only found at a pH ≥ 7.79 pH. On the bottom-sides of the tiles, differences in pH tolerance between species were significant yet relatively minor. Similar to our results, Doropulous et al (2013) identified members of the genera *Titanoderma* sp. and *Hydrolithon* spp. as the most CO_2_ sensitive and *P. onkodes* the least sensitive CCA taxa, while *H. reinboldii* appeared more sensitive in our field than in their mesocosm study^6^. Our observed differences in CO_2_ sensitivity between species were not attributable to any obvious features. The most sensitive taxa in the field, namely *Titanoderma prototypum* and *Hydrolithon reinboldii*, belong to two different subfamilies (subfamily Lithophylloideae and Mastophorideae), while the most pH tolerant taxa (*Lithoporella melobesioides, Paragoniolithon conicum* and *Porolithon onkodes*) also belong to the subfamily Mastophorideae. Differences between species were also not attributable to obvious morphological features (e.g., taxa forming thin or thick crusts once mature^6^,^20^, or hypothallial structures). However all specimens in our study were still thin crusts (<0.5 mm), probably due to their young age and the heavy grazing regime on the upward facing surfaces.

Our study design facilitated the estimation of CCA response curves to rising CO_2_ *in situ*, after many months of exposure. The data show that the declines in cover along the pH gradient were log-linear in both high and low light environments, with steeper losses at the lower compared to the higher end of the CO_2_ range. There was however no discrete physiological tipping point at any particular level. The data therefore strongly suggest that significant declines in CCA communities are already unfolding in both tropical and temperate waters, and that CCA will continue to decline in the future due to the ongoing rapid increases in CO_2_.

The greater variability in seawater carbonate chemistry at CO_2_ seeps compared to future high CO_2_ oceans^31^ makes it impossible to use these settings to define exact lower limits for pH tolerance. Recent studies have shown that rates of pH change may contribute to affect the OA vulnerability of CCA, and that acclimatisation to variable pH does not make CCA more tolerant of declining pH^32^. Nevertheless, as coastal waters can also experience substantial pH variability^33^, and as exposure to other potential co-limiting factors such as grazing are far more realistic in the field than in the laboratory, lower pH limits are probably best derived from field data, albeit with caution. Furthermore, the CCA on our tiles were exposed to their pH environment since settlement and throughout their life, providing more realistic scope for acclimatisation than short-term laboratory experiments do^34^. At the PNG seeps, there was a steep transition from tiles with CCA to those without CCA at a pH of ~7.8 (~660 μatm *p*CO_2_, Ω_Arag_ = 2.8), a value that has been forecasted for the middle of this century by many emissions scenarios. At this level, shaded and light exposed CCA communities were reduced to 37% and 54% of cover at a pH of 8.0. At pH levels of ≤7.7 (~860 μatm *p*CO_2_, Ω_Arag_ = 2.2), a value that may be reached in the second half of this century, cover was down to ~3% of that at control sites. Similarly, CCA was absent on seagrass blades at ≤7.7 pH at temperate CO_2_ seeps, suggesting this apparent lowest limit for CCA *in situ* may not be restricted only to tropical Indo-Pacific CCA communities^14^. These lower limits match those of the loss in reef development at the PNG seep sites, where reef communities are marginal at 7.8 pH, while at a pH ≤ 7.7, coral reef development ceases with only a few individual coral colonies persisting below this level^15^. Whether there is a causal relationship between these limits for the CCA communities and for reef development at the PNG seeps remains unknown. However, given the important ecological role of CCA, it is likely that their losses due to anthropogenic CO_2_ emissions would lead to profound ecological changes in many aspects of benthic marine ecosystems.

## Methods

### Seawater Chemistry

The study was conducted at three island fringing reefs in Milne Bay Province, Papua New Guinea: Dobu, Esa'Ala and Upa Upasina (latitude 9°45′–9°49′ S, longitude 150°49′–150°52′ E), which are located on an active tectonic fault line where the continental plates of Australia and the Solomon Islands are spreading apart. Each reef contains an area where almost pure (~99%) volcanic CO_2_ is seeping in shallow water (<5 m depth) from the seafloor, and a control area ~0.5 to 2 km away from each seep with similar geomorphological settings that is not exposed to CO_2_ seepage^15^. All sites are very similar in their environmental conditions, including temperature, salinity, light and currents^15^.

The seawater chemistry data for the reef sites along the transects are published in Fabricius et al. (2014)^24^. Above each of the numbered tiles, seawater samples were also repeatedly collected during four ~2-week long visits between 2011 and 2013. After returning to the boat, temperature and pH were measured immediately with a pH electrode following standard procedures^34^. A total of 1134 samples were analysed for pH, with a median of 8 samples per tile (range: 4–23), and a subset were analysed for salinity (Mettler handheld salinity meter). Another subset of 728 samples (median: 5 per tile, range: 3–18) was preserved with mercury chloride in 250 ml polycarbonate bottles for later determination of other seawater carbonate parameters. Of these, 366 samples were analysed for combined total alkalinity (TA) and dissolved inorganic carbon (DIC) with a Vindta 3C (Marianda), the remaining samples were analysed for TA with a Metrohm 855 automated open cell potentiometric titrator^35^. The remaining seawater carbonate parameters were calculated from the pH, TA, salinity and temperature data with the R program Seacarb v2.4.8 (Lavigne, H. & Gattuso, J. P., http://cran.r-project.org/web/packages/seacarb/index.html).

Of the seawater chemistry variables, the medians and percentiles of pH (converted to total scale), DIC, partial pressure of CO_2_ (*p*CO_2_), and the saturation state of calcite and aragonite (Ω_Calc_ and Ω_Arag_) were all highly correlated (correlation coefficients ranging from 0.81 to 0.97), and median pH was chosen as a proxy for changes in all these variables as predictor variable for the models. TA was less variable and hence more weakly correlated to the other carbonate chemistry variables (correlation coefficients to the other variables: 0.39 to 0.57).

### Biotic data

Photo-transects (85 in total, 10 m long, 0.5 m wide, one image every 0.5 m) were used to assess total CCA cover. At Upa-Upasina, 20 and 25 transects were investigated at the High CO_2_ and Control site, respectively; at the other reefs, the number of transects was 10 per site. Photo analysis followed Jonker et al. (2008), determining the benthos substrata to the highest possible taxonomic resolution underneath 5 fixed points in each image^36^.

A total of 120 settlement tiles (11.5 × 11.5 × 0.3 cm, made of polyvinyl chloride with surfaces roughened by sand paper) were deployed in December 2011. There was one Control site at each reef, as well as two High CO_2_ sites at Dobu and Upa-Upasina, and one High CO_2_ site at Esa'Ala. At each site, 15 tagged tiles were distributed over an area of ~200–400 m^2^ at 3 m depth. Each tile was secured horizontally ~2 cm above the reef to a tagged base plate. All tiles were first collected after five months (May 2012; all 120 plates were still in place). While being kept submerged in sea water at all times, the top- and bottom-sides were photographed, and tiles were returned to their original location within a few hours of collection. After 13 months deployment (January 2013), the tiles were again collected, with 116 of the tiles still in place. Tiles were again photographed (photographs from 3 of the 8 sites of the 13 months census were lost), rinsed in fresh water, dried, and stored for transport.

To assess benthos cover of the tiles, the images were digitally adjusted for tilt, size, colour and contrast. Grid lines (7 × 7) were overlaid, with the outermost lines crossing 0.5 cm from the tile edges. Substrata were recorded for the 49 grid points, distinguishing 6 and 14 categories for the top- and bottom-sides of the tiles, respectively. Additionally, CCA were identified to highest taxonomic level possible by inspecting conceptacles and other taxonomic features, following Adey et al. (1982)^1^. For each CCA taxon, the percent cover was visually estimated using a dissecting microscope and a grid as visual guide. Initial inspection showed the top-side communities were dominated (>98%) by the CCA species *Porolithon onkodes*. On the bottom-sides, eight species, one group of early successional crusts that were too young or too poorly developed to show taxonomic features, and one group of unidentified CCA were distinguished.

Ratios of CCA cover (High CO_2_/Controls, Fig. 2) were estimated with generalised linear models (GLMs) with log link function and quasipoisson distribution^37^ and the two predictors pH and reefs. The final analysis included only pH as reef effects were non-significant. GLMs were also used to estimate the effect of pH on the cover of individual species on the tiles, and to predict their cover values for pH conditions at 7.8 and 8.0 (Fig. 4). Tile identity was used as the error term. GLMs were then used to investigate the effects of orientation (top- vs. bottom-sides), and succession (5 vs. 13 months), as well as pH, on total CCA cover (Table 1). All analyses used the statistical package R (R Development Core Team, version 3.0.2), including the R packages Seacarb, beanplot and mgcv.

## Supplementary Material

Supplementary Information Supplementary Information

## Figures and Tables

**Table 1:** Effects of pH, orientation (top-side with high light and intense grazing, vs bottom side with low light and little grazing), and time (5 vs 13 months of deployment) on CCA cover on the tiles (Fig. 4 - top row). Generalized linear model, and backward elimination of non-significant interaction terms

	Df	Deviance	F	P
NULL	264	4451.5		
pH	1	1901.7	326.5	<0.001
Orientation	1	256.8	44.10	<0.001
Time	1	0.600	0.103	0.748
Tile	65	930.0	2.457	<0.001
pH: Orientation	1	118.1	20.28	<0.001
pH: Time	1	37.84	6.498	0.012
