# Supplementary material for: In situ changes of tropical crustose coralline algae along carbon dioxide gradients
Source: Sci Rep. 2015 Apr 2;5:9537. doi: 10.1038/srep09537 (PMC5381686; doi:10.1038/srep09537)
Supplement: Supplementary Information [file srep09537-s1.pdf]

Supplementary Information

In situ changes of tropical crustose coralline algae along carbon dioxide gradients

K. E. Fabricius, A. Kluibenschedl, L. Harrington, S. Noonan, G. De'ath

Supplementary Table S1: Seawater chemistry at the tiles from the three Control sites and five High CO_2 ('Seep') sites. Displayed are the number of tiles (N), and the site medians (and 5th and 95th percentiles) of the median values per tile. Abbreviations: TA = total alkalinity (in µmol equivalents kg^{-1}), pCO_2 = partial pressure of CO_2 in the seawater (µatm), Ω_{Arag} and Ω_{Calc} = aragonite and calcite saturation state, DIC = dissolved inorganic carbon (in µmol kg^{-1}).

			pH		TA		*p*CO2	
Reef	Site	N	med	(05th, 95th)	med	(05th, 95th)	med	(05th, 95th)
Dobu	Control	15	7.99	(7.97, 8.10)	2235	(2219, 2262)	442.4	(304.3, 469.4)
	Seep 1	14	7.67	(7.19, 7.95)	2290	(2274, 2309)	781.7	(535.7, 2656)
	Seep 2	14	7.73	(7.39, 7.92)	2291	(2268, 2313)	1183	(684.9, 1962)
Esa'Ala	Control	15	8.03	(7.97, 8.08)	2260	(2253, 2280)	309.5	(257.8, 483.8)
	Seep 1	15	8.02	(7.96, 8.07)	2289	(2287, 2293)	290.8	(275.5, 291.7)
Upa-U.	Control	15	8.00	(7.92, 8.09)	2271	(2223, 2336)	342.5	(301.5, 516.9)
	Seep 1	15	7.83	(7.41, 7.98)	2290	(2242, 2365)	634.8	(491.5, 1196)
	Seep 2	13	7.84	(7.62, 7.97)	2309	(2275, 2338)	604.5	(466.4, 778.7)

		Ω_{Calc}		Ω_{Arag}		DIC	
Reef	Site	med	(05th, 95th)	med	(05th, 95th)	med	(05th, 95th)
Dobu	Control	5.31	(4.80, 6.55)	3.56	(3.20, 4.40)	1927	(1851, 1945)
	Seep 1	3.49	(1.47, 4.50)	2.32	(0.98, 2.98)	2113	(2026, 2250)
	Seep 2	2.87	(1.79, 4.11)	1.92	(1.20, 2.74)	2147	(2062, 2231)
Esa'Ala	Control	6.73	(5.09, 7.24)	4.52	(3.41, 4.85)	1873	(1829, 1965)
	Seep 1	7.27	(7.12, 7.30)	4.89	(4.79, 4.93)	1863	(1849, 1863)
Upa-U.	Control	6.20	(4.79, 6.77)	4.11	(3.20, 4.57)	1925	(1869, 1967)
	Seep 1	3.93	(2.82, 5.03)	2.61	(1.88, 3.36)	2047	(1980, 2196)
	Seep 2	4.31	(3.89, 5.03)	2.87	(2.61, 3.36)	2063	(1986, 2115)

Supplementary Table S2: Difference in seawater chemistry values (medians) between Seep and Control sites (Fig. 1), and between the three reefs. Analysis of deviance tables, based on generalized linear models with log-link function and quasipoisson distribution.

	**Df**	**Deviance**	**Residual Df**	**Residual Deviance**	**F**	**P**
Response: pH						
NULL	115	0.3959				
Site2	1	0.1469	114	0.2491	126.1	<0.0001
Reef	2	0.1182	112	0.1309	50.71	<0.0001
Response: Omega calcite						
NULL	115	56.60				
Site2	1	15.80	114	40.79	120.4	<0.0001
Reef	2	25.64	112	15.15	97.69	<0.0001
Response: Omega Aragonite						
NULL	115	38.24				
Site2	1	10.55	114	27.69	119.1	<0.0001
Reef	2	17.48	112	10.21	98.65	<0.0001
Response: pCO_2						
NULL	115	22142				
Site2	1	7430.4	114	1471	123.1	<0.0001
Reef	2	8669.5	112	6041.6	71.80	<0.0001
Response: Total Alkalinity						
NULL	115	32.56				
Site2	1	18.77	114	13.75	213.0	<0.0001
Reef	2	3.877	112	9.878	21.99	<0.0001
Response: DIC						
NULL	115	690.7				
Site2	1	281.7	114	409.0	224.0	<0.0001
Reef	2	268.4	112	140.6	106.7	<0.0001

Supplementary Table S3: Percent cover of crustose coralline algae (CCA) and other benthic groups on the reef and on the top- and bottom-sides of the settlement tiles at the High CO_2 and Control sites (Fig. 2). Ratios and their 95% confidence intervals are back-transformed logged values. Numerous groups of invertebrate phyla complement the values on the bottom-sides of the tiles to 100%. Differences between ratios are significant at the 5% level if the range between upper and lower confidence intervals of the ratios does not include the value 1.0.

	% Control	(upper, lower 95% CI)	% High CO_2	(upper, lower 95% CI)	Ratio	(upper, lower 95% CI)
**Photo transects**						
CCA	6.65	(4.43, 8.53)	2.22	(1.48, 3.34)	0.33	(0.21, 0.54)
Unoccupied	30.2	(25.1, 35.3)	24.1	(20.1, 29.0)	0.80	(0.63, 1.02)
**Top-sides tiles – 5 months**						
CCA	38.1	(29.7, 43.3)	6.4	(5.0, 8.15)	0.17	(0.13, 0.22)
Unoccupied	22.8	(20.3, 27.4)	36.0	(32.1,40.4)	1.58	(1.27, 1.96)
Green filamentous algae	18.2	(15.5, 22.8)	23.3	(19.9, 27.4)	1.28	(0.97, 1.70)
Turf algae	17.8	(14.5, 24.3)	26.0	(21.2,31.8)	1.46	(1.01, 2.12)
Cyanobacteria	2.32	(1.89, 3.67)	6.9	(5.59,8.51)	2.97	(1.79, 4.91)
Macroalgae	0.84	(0.58, 1.63)	1.7	(1.19,2.51)	2.07	(0.96, 4.44)
**Top-sides tiles – 13 months**						
CCA	29.6	(23.1, 35.6)	11.1	(8.65, 14.2)	0.37	(0.27, 0.51)
Unoccupied	16.9	(14.2, 20.0)	11.8	(9.99, 14.0)	0.70	(0.55, 0.89)
Green fil. algae	14.3	(12.0, 18.5)	21.7	(18.2, 25.9)	1.52	(1.11, 2.08)
Turf algae	31.7	(25.9, 40.7)	33.4	(27.3, 40.9)	1.05	(0.76, 1.46)
Cyanobacteria	3.4	(2.46, 6.65)	9.2	(6.62, 12.9)	2.70	(1.29, 5.66)
Macroalgae	4.1	(3.13, 7.50)	12.7	(9.60, 16.8)	3.07	(1.59, 5.92)
**Bottom-sides tiles – 5 months**						
CCA	23.5	(17.3, 28.5)	5.9	(4.33, 7.95)	0.25	(0.17, 0.36)
Unoccupied	21.1	(17.7, 26.3)	19.5	(16.3, 23.2)	0.92	(0.70, 1.22)
Turf algae	17.3	(15.2, 20.8)	21.2	(18.7, 24.2)	1.23	(0.98, 1.54)
Cyanobacteria	0.7	(0.61, 2.40)	23.7	(20.1, 27.9)	33.1	(9.77, 112.1)
Macroalgae	0.1	(0.10, 2.05)	3.7	(2.47, 5.55)	26.0	(1.76, 385.6)
**Bottom-sides tiles – 13 months**						
CCA	21.4	(16.1, 25.9)	6.2	(4.69, 8.26)	0.29	(0.21, 0.41)
Unoccupied	7.6	(4.88, 11.3)	4.4	(2.84, 6.92)	0.58	(0.32, 1.06)
Turf algae	7.6	(5.65, 10.7)	7.2	(5.35, 9.74)	0.95	(0.60, 1.49)
Cyanobacteria	0.7	(0.58, 2.00)	25.1	(21.5, 29.2)	36.8	(12.4, 109.6)
Macroalgae	0.3	(0.28, 2.42)	22.8	(18.6,28.0)	67.2	(9.31, 483.3)

Supplementary Table S4: Relationship of specific taxonomic groupings of crustose coralline algae (CCA) on settlement tiles to pH. The first column shows the percentage of tiles with the taxon present. Consecutive columns show estimated cover at both 8.0 and 7.8 pH (with upper and lower 95% confidence intervals), the ratios of these estimates, the slope of change, and the significance of difference between the two pH levels (Fig. 4). The first four rows shows total cover of CCA (all taxa combined) on the top- and bottom-sides of the tiles after 5 and 13 months of deployment (5 M, 13 M). The rows below show estimates of the cover of specific CCA taxa on the bottom-sides after 13 months.

	% Present	Est. cover pH 8.0	(Upper, lower 95% CI)	Est. cover pH 7.8	(Upper, lower 95% CI)	Ratio cover pH 7.8/8.0	Slope	t	P
Total cover Top (5 M)	92.0	25.9	(21.8, 30.7)	9.30	(6.72, 12.9)	0.36	5.11	6.092	<0.001
Total cover Top (13 M)	95.7	27.6	(22.9, 33.4)	14.8	(12.2, 18.0)	0.54	3.12	5.428	<0.001
Total cover Bottom (5 M)	74.1	17.9	(15.4, 20.8)	4.66	(3.29, 6.59)	0.26	6.73	7.721	<0.001
Total cover Bottom (13 M)	80.4	16.3	(13.8, 19.3)	6.05	(4.44, 8.22)	0.37	4.97	6.238	<0.001
Unidentified spp.	28.6	2.77	(2.15, 3.57)	0.80	(0.47, 1.38)	0.29	6.19	4.524	<0.001
Successional spp.	59.8	6.52	(5.02, 8.46)	2.32	(1.43, 3.77)	0.36	5.16	4.117	<0.001
Unidentified sp. 1	18.6	0.77	(0.46, 1.32)	0.12	(0.021, 0.62)	0.15	9.55	2.345	0.021
Titanoderma prototypum	31.3	0.82	(0.51, 1.33)	0.29	(0.11, 0.72)	0.35	5.29	2.235	0.027
Hydrolithon reinboldii	36.6	1.01	(0.65, 1.58)	0.37	(0.16, 0.83)	0.36	5.05	2.398	0.018
Neogoniolithon (cf. *frutescens*)	59.8	0.78	(0.50, 1.21)	0.31	(0.14, 0.66)	0.39	4.67	2.319	0.022
Lithoporella melobesioides	24.1	0.75	(0.46, 1.22)	0.35	(0.17, 0.74)	0.47	3.82	1.923	0.057
Paragoniolithon conicum	48.2	2.44	(1.79, 3.32)	1.38	(0.92, 2.09)	0.57	2.84	2.564	0.012
Porolithon onkodes (Low)	5.4	0.06	(0.02, 0.16)	0.03	(0.005, 0.13)	0.48	3.64	0.853	0.396
Lithophyllum kotschyanum	4.5	0.29	(0.09, 0.98)	0.002	(0.000, 1.70)	0.01	26.3	1.703	0.091
